# Health insurance of rural/township schoolchildren in Pinggu, Beijing: coverage rate, determinants, disparities, and sustainability

**DOI:** 10.1186/1475-9276-7-23

**Published:** 2008-11-03

**Authors:** Jane M Zhu, Yiliang Zhu, Rui Liu

**Affiliations:** 1 Harvard Medical School, 260 Longwood Ave, Rm. 233 Boston, MA 02115, USA; 2 Sanford Institute of Public Policy, Duke University, P.O. Box 99712, Durham, NC 27708, USA; 3 Formally: Fudan University School of Public Health, Shanghai, PR China; 4 Department of Epidemiology and Biostatistics, College of Public Health, University of South Florida, 13201 Bruce B. Downs Blvd. MDC 56, Tampa, Florida 33612-3805, USA; 5 Health Services and Policy Analysis, 140 Warren Hall #7360, University of California-Berkeley, Berkeley, California 94720, USA; 6 Formally: Beijing University Guanghua School of Management, Haidian District, Beijing, 100871, PR China

## Abstract

**Background:**

As China re-establishes its health insurance system through various cooperative schemes, little is known about schoolchildren's health insurance. This paper reports findings from a study that examined schoolchildren's insurance coverage, disparities between farmer and non-farmer households, and effects of low-premium cooperative schemes on healthcare access and utilization. It also discusses barriers to sustainable enrollment and program growth.

**Method:**

A survey of elementary school students was conducted in Pinggu, a rural/suburban district of Beijing. Statistical analyses of association and adjusted odds ratio via logistic regression were conducted to examine various aspects of health insurance.

**Results:**

Children's health insurance coverage rose to 54% by 2005, the rates are comparable for farmers' and non-farmer's children. However, 76% of insured farmers' children were covered under a low-premium scheme protecting only major medical events, compared to 42% among insured non-farmers' children. The low-premium schemes improved parental perceptions of children's access to and affordability of healthcare, their healthcare-seeking behaviors, and overall satisfaction with healthcare, but had little impact on utilization of outpatient care.

**Conclusion:**

Enrolling and retaining schoolchildren in health insurance are threatened by the limited tangible value for routine care and low reimbursement rate for major medical events under the low-premium cooperative schemes. Coverage rates may be improved by offering complimentary and supplementary benefit options with flexible premiums via a multi-tier system consisting of national, regional, and commercial programs. Health insurance education by means of community outreach can reinforce positive parental perceptions, hence promoting and retaining insurance enrollment in short-term.

## Background

By the 1970s, nearly all urban Chinese population and 85% rural residents were covered under a health insurance scheme[1]. Market-oriented reform in the following decades witnessed the disintegration of the healthcare system and the disappearance of the public insurance systems[2]. By 2003, insurance coverage fell to 54–55% in urban population with only 12% of the poorest fifth covered[3,4], while 79% (640 million) rural residents were without insurance due to the dissolution of agricultural communes that had served as the primary payer[2,3,5]. In the meantime, out-of-pocket medical costs climbed steadily[2,6], healthcare utilization declined[3], and barriers to healthcare rose, particularly for the poor and the rural [7,8].

In 1998 the Chinese government began to establish a basic health insurance scheme (BHIS) for registered urban workers and retirees[9]. The cooperative BHIS does not, however, cover children or other dependents[9,10]. In 1994 the government began to pilot a new rural cooperative medical system (RCMS) in rural areas[11], expanding the program to 310 counties by 2004[5,12] and aiming to cover the entire rural population by 2010. Only farmers are eligible for RCMS and enrollment is voluntary in unit of a household. As of 2006, households, local, and central governments each contributed no less than 20 yuan (RMB) per enrollee[13]. Amid these fundamental reforms, health insurance access and coverage of schoolchildren is largely unknown[8]. Except for a few earlier studies on children's health insurance coverage using data from the China Health and Nutrition Survey prior to 1997[6,8], studies on healthcare access, outcomes, and disparities between urban and rural populations generally have not examined children [14-16]. For instance, the 2003 Third National Health Services Survey (NHSS) remained non-specific to the country's 270 million children[3]; another study by Xu et al [4] only considered age-group insurance coverage for urban population based on the 2003 NHSS data.

As China adopts national and regional cooperative schemes to re-establish a national health insurance system, achieving and sustaining a high enrollment rate are a benchmark for program success. It is thus critical to identify barriers to enrollment, uncover disparities among rural and urban populations, and evaluate perceived and tangible benefits of existing cooperative schemes. Based on a survey of elementary schoolchildren, this paper focuses on disparate health insurance coverage among farmers' and non-farmers' children, along with their access to and utilization of healthcare under various insurance schemes. It also discusses potential threats to sustainable insurance enrollment, and recommends measures for program improvement.

## Methods

### Study Setting

Pinggu is a mountainous district in eastern Beijing; over 75% of its 397,000 residents are farmers and 60% of land area agricultural. The area represents a growing segment of rural China that is in close proximity to major cities and is undergoing rapid socioeconomic transition. The BHIS was established there in 2001 and the RCMS in 2004. Beginning in the 1990s, a *Student Safety and Health Insurance *(SSHI) program was introduced through local school administrations in partnership with commercial vendors. The SSHI charges an annual premium of 60 to a hundred some yuan (RMB), reimburses partially medical expenses incurring from major events such as surgery and hospitalization. In September 2004, the local Red Cross, municipal Education Commission, and Bureau of Hygiene and Health jointly established a Children's Hospitalization Cooperative Fund (CHCF), which offers a not-for-profit, cooperative scheme to all local schoolchildren. At a 50-yuan annual premium, CHCF progressively covers up to 50% medical expenses, with a cap of 80,000 yuan/year, for hospitalization, surgery, and special treatments such as chemotherapy and dialysis. Schoolchildren thus have choices among SSHI, CHCF, RCMS, or commercial schemes.

### Survey

Four primary schools were selected from 108 by Pinggu's Education Bureau. All first and fourth graders were invited. A questionnaire was distributed in class, and filled by a parent or guardian of each class-attending student. Of 611 questionnaires distributed, 490 (80%) were returned. The questionnaire collected demographic and socioeconomic information, child's health status, insurance coverage, healthcare access and utilization, and parental perceptions of the health insurance system and healthcare system. Data on insurance included the child's insurance status, history, premium, and benefits. Parental perception about insurance and healthcare systems consisted of satisfaction, expectations of service, and perceived barriers.

### Statistical Analysis

S-Plus 6.2 ^® ^(Insightful, Seattle) was used for analysis. Descriptive statistics for selected demographic factors and their association with insurance status were reported. Adjusted odds ratios via logistic regression, their confidence interval, and likelihood ratio tests were calculated to evaluate potential determinants to insurance coverage. Disparities in care access and utilization between farmers' and non-farmers' children were analyzed through chi-squared tests.

## Results

### Sample Characteristics

Table 1 summarizes household characteristics. Of 494 participating children in total, including four pairs of twins, 55.0% (n = 260) were female and 45.0% (n = 213) were male, 21 did not report gender. There were 319 (66.0%) one-child households, while 164 (34.0%) households had two or more children. The sample children were generally healthy, with 88.6% of parents reporting children in very good or excellent health.

**Table 1:** Children's Characteristics and Health Insurance Status

**Variable**	**Total^1^** **% (N)**	**Insurance Rate^2^**		**p-value^3^**
			
		**% (n)**	**95% CI**	
**Sample children**	100 (494)	54.0 (249)	49.5 – 58.5	
**Child's age (years)**				0.495
5–8	35.6 (170)	51.9 (81)	44.1 – 59.8	
9–12	64.4 (308)	55.8 (164)	50.1 – 61.5	
**Child's gender**				0.792
Male	45.0 (213)	55.6 (110)	48.6 – 62.5	
Female	55.0 (260)	53.8 (133)	47.6 – 60.1	
**Number of children in household**				0.012
One	66.0 (319)	58.0 (178)	52.5 – 63.5	
Two	27.1 (131)	50.8 (61)	41.9 – 59.8	
Three or more	6.9 (33)	29.6 (8)	12.4 – 46.9	
**Parental rating of child's health**				0.142
Average or below	11.4 (55)	44.0 (21)	30.2 – 57.8	
Very good	47.2 (227)	52.4 (110)	45.6 – 59.1	
Excellent	41.4 (199)	58.6 (112)	51.7 – 65.6	
**Annual per capita income (yuan)**				0.118
1^st ^quartile (< 1,667)	29.3 (137)	50.0 (62)	41.2 – 58.8	
2^nd ^quartile (1,667 – 3,125)	24.1 (113)	48.6 (51)	39.0 – 58.1	
3^rd ^quartile (3,126 – 6,667)	21.6 (101)	55.8 (53)	45.8 – 65.8	
4^th ^quartile (> 6,667)	25.0 (117)	62.9 (73)	54.1 – 71.7	
**Highest education of household head**				0.002
Completed primary school or below	6.0 (29)	33.3 (7)	13.2 – 53.5	
Completed middle school	33.3 (162)	44.5 (69)	36.7 – 52.3	
Completed high school	25.3 (123)	58.6 (68)	49.7 – 67.6	
Post-secondary	35.4 (172)	63.3 (105)	55.9 – 70.6	
**Parental insurance status**				<0.001
At least one insured	53.1 (246)	74.4 (177)	68.8 – 79.9	
None insured	46.9 (217)	33.3 (68)	26.9 – 39.8	
**(1^st^:2^nd^) Parental Occupation**				0.044
Both gov./state employees	20.4 (96)	61.3 (57)	51.4 – 71.2	
Self-empl./private:self/priv/gov/state	27.6 (131)	45.5 (56)	36.7 – 54.3	
One or both farmer	41.0 (195)	58.0 (105)	50.8 – 65.2	
Non-farmer, unemployed	11.0 (53)	46.9 (23)	33.0 – 60.9	

1. Sample distribution (percentage) irrespective of insurance information

2. Missing data excluded

3. Association with insurance status was tested using Chi-squared statistic

Farming was the single-most common occupation, with 34.5% (n = 164) households having two farming-parents, and 6.5% (n = 31) households having one farming-parent, respectively. About 11% (n = 53) households had at least one unemployed parent. Fifteen percent households had annual incomes over 35,000 yuan, but three-quarters of both-farmer-parent households earned 10,000 yuan or less. The majority of household heads (60.7%) had completed high school or beyond, and over half (53.1%) reported having health insurance of their own.

### Rate of Insurance Coverage and Determinants

Overall, 54% (n = 249) children had some type of health insurance, although the coverage was uneven across a number of factors (table 1). Decisions to buy insurance for a child was not associated with gender (p = 0.79), age (p = 0.50), or general health (p = 0.14). Household per capita income did not significantly influence the decision either (p = 0.12). However, children of an insured parent were more than twice as likely to have insurance as those of uninsured parents (74% vs. 33%, p < 0.001). Households of 1–2 children were more likely to have insurance for the participating child than those with more children (53% vs. 30%, p = 0.012). While only 33% of households in the lowest education group enrolled their children in an insurance program, the rate rose steadily to 63% among households in the post-secondary education group (p = 0.002). Interestingly, the coverage rate among farming households (with at least one farming parent) was comparable to that of government/state enterprise employed parents (58% vs. 61%), but higher than households of other occupations.

The decision to enroll a child in an insurance scheme results from the interplay of healthcare need and cost-benefit considerations. We used a logistic regression model to evaluate the impact of potential surrogates of cost and benefit on the decision to enroll, and present adjusted odds ratios (OR) for insurance in Table 2. On the affordability (cost) end, families with 3+ children were only 38% as likely to have insurance for a child as families with 1–2 children (OR = 0.38, CI = 0.11–1.40, p = 0.002); farmer's households (OR = 7.22) and those with an unemployed parent (OR = 2.57) were more likely to buy insurance than households of non-unemployed, non-farmer parents (p-value = 0.03). It is noteworthy that the rate of coverage among households that perceived insurance to be affordable was comparable to that among households unable to afford insurance (OR = 1.07). In contrast, households with neutral perceptions about insurance affordability or just somewhat positive were much less likely to have insurance (OR = 0.47, 0.65, respectively, p < 0.001). On the perceived "benefit" end, children of uninsured parents were much less likely to have insurance than those with an insured parent (OR = 0.01, p < 0.001); parents who had positive opinion about the insurance system were more than 3 times as likely to buy insurance for the child as those who one level less positive in terms of satisfaction ("dissatisfied", "neutral/somewhat satisfied", "satisfied") (OR = 3.17, p < 0.001). Parental educational level, as a multi-faceted factor related to affordability, as well as knowledge and perceptions about insurance, did not influence a child's insurance status if the parent was also insured, but played a promotional role if the parent was uninsured: parents of a given level of education were 2.59 times as likely to enroll a child as their counterpart whose education was one level lower (OR = 2.59, p-value < 0.001).

**Table 2:** Determinants of and Barriers to Children's Health Insurance

**Determinant**	**Insured vs. Uninsured**		
	
	**Adjusted OR**	**95% CI^1^**	**p-value^2^**
**Affordability for insurance premium**			<0.001
Unaffordable	1		
Neutral	0.47	(0.20, 1.13)	
Somewhat affordable	0.65	(0.27, 1.56)	
Affordable	1.07	(0.46, 2.52)	
**Satisfaction about insurance system **^3^	3.17	(2.10, 4.79)	<0.001
**Number of children in household**			0.002
One or two	1		
Three or more	0.38	(0.11, 1.40)	
**Parental Occupation**			0.030
Both gov./state enterprise employees	1		
Non-farmer & at least one self employed	1.31	(0.63, 2.71)	
One or both farmer	7.22	(2.96, 17.64	
Non-farmer & at least one unemployed	2.57	(0.91, 7.24)	
**Insurance status of any parents**			<0.001
Insured	1		
Uninsured	0.01	(0.001, 0.04)	
**Education of *uninsured *household head**^4^	2.59	(1.56, 4.31)	<0.001

1. Approximate confidence interval derived as mean ± 1.96*standard error.

2. P-value based on likelihood ratio test for the overall effect of each factor.

3. Ordinal scale was replaced by continuous ratio in increasing satisfaction: dissatisfied = 1, neutral = 2, satisfied = 3

4. Ordinal scale was replaced by continuous ratio in increasing education level (see Table 1); effects applicable only to households with no insured parent

### Disparity in Coverage

Although disparity in children's coverage was muted between farmers and non-farmers' households, it existed with respect to the type of insurance programs. Compared with low-premium schemes RCMS, CHCF, and SSHI, commercial policies required an annual premium as high as 10,000 yuan, with a median of 1,000 yuan. Farmers' children were enrolled overwhelmingly in low-premium schemes (i.e. RCMS, CHCF, or SSHI), rather than commercial options (75.7% vs. 24.3%); in contrast, 58% and 40% of children in the "non-farmer & employed" and "non-farmer & unemployed" occupational groups, respectively, were covered under a commercial policy (χ^2 ^= 25.3, p = 0, Table 3).

**Table 3:** Primary Insurance Scheme^1 ^by Parental Occupation^2^

**Parents**	**Commercial**	**CHCF**	**SSHI**	**RCMS**	**Total^3^**
**Non-farmer & employed**	64(58.2%)	17(15.5%)	29(26.4%)	NA	110
**One or both farmer**	25(24.3%)	14(13.6%)	53(51.5%)	11(10.7%)	103
**Non-farmer & unemployed**	9(39.1%)	7 (30.4%)	7 (30.4%)	NA	23

1. Fifteen children under a commercial policy also had a CHCF/SSHI; five had both RCMS and SSHI or CHCF and SSHI; primary scheme was determined in the order of commercial, RCMS, CHCF, and SSHI;

2. Fourteen children's insurance type or parental occupation cannot be determined

3. Combining SSHI, CHCF, and RCMS into one category of low premium, insurance scheme differs significantly between farmer and non-farmer households (χ^2 ^= 25.25, DF = 2, p-value = 0)

### Care Access and Utilization

Did insurance coverage, particularly the cooperative, low-premium schemes, improve schoolchildren's access to and utilization of care? Table 4 presents results from our comparison of three groups: uninsured, insured under low-premium schemes, and insured under commercial schemes. We found that insurance coverage generally improved access to care. Among the group covered under a low-premium or cooperative scheme, 43% parents perceived little difficulty in healthcare access compared with 15% who had difficulty; among parents whose child was under a commercial scheme, the percentage was 51% vs. 8%; for uninsured children, only 24% of parents perceived no difficulty, while 17% did. This pronounced difference (p < 0.001) suggested that both commercial and cooperative schemes improved parental perceptions of healthcare access. However, 24% parents whose children were enrolled in a low-premium scheme felt healthcare to be unaffordable, compared with 14% and 18% under commercial schemes and uninsured, respectively. Conversely, 58%, 62%, and 52% of parents in the low-premium, commercial, and uninsured groups, respectively, felt healthcare to be affordable. These group differences (p = 0.08) implied that the low-premium schemes only provided limited relief of financial burden despite perceptions of improved access.

**Table 4:** Disparities in Healthcare Access and Utilization

	**Commercial**	**Low-Premium**	**Uninsured**	**p-value**
**Difficulty in access**	n = 98	n = 134	n = 198	<0.001
No	51.0%(50)	43.3%(58)	24.2%(48)	
Neutral	40.8%(40)	41.8%(56)	58.6%(116)	
Yes	8.2%(8)	14.9%(20)	17.2%(34)	

**Care Affordability**	n = 102	n = 135	n = 202	0.077
Affordable	61.8%(63)	57.8%(78)	52.0%(105)	
Neutral	24.5%(25)	18.5%(25)	29.7%(60)	
Unaffordable	13.7%(14)	23.7%(32)	18.3%(37)	

**Delay in seeking care**^1^	n = 78	n = 106	n = 161	0.105^2^
Yes	17.9%(14)	13.2%(14)	23.6%(38)	

**Foregone care**	n = 96	n = 133	n = 192	0.009
Yes	11.5%(11)	14.3%(19)	24.5%(47)	

**Outpatient Visits**	n = 101	n = 140	n = 205	0.796
None	18.8%19)	24.3%(34)	19.5%(40)	
1–2	59.4%(60)	59.3%(83)	57.6%(118)	
3–5	16.8%(17)	12.1%(17)	17.1%(35)	
>5	5.0%(5)	4.3%(5)	5.9%(12)	

**Satisfaction with healthcare**	n = 99	n = 139	n = 198	0.067^3^
Satisfied	21.2%(21)	25.2%(35)	12.2%(24)	
Somewhat satisfied	40.4%(40)	41.0%(57)	43.4%(86)	
Neutral	17.2%(17)	15.8%(22)	23.7%(47)	
Unsatisfied	21.2%(21)	18.0%(25)	20.7%(41)	

1. Only those reported illness were included

2. Difference between the low-premium and uninsured was more pronounced with χ^2 ^= 3.77, df = 1, p-value = 0.05

3. Comparison of insured (commercial and coop combined) and uninsured resulted in χ^2 ^= 10.915, df = 3, p-value = 0.012

These differential perceptions of access and affordability were also mirrored in the incidence of delayed or forgone care. When ill, 13.2% children under a low-premium scheme delayed care-seeking, compared with 17.9% of those under a commercial scheme and 23.6% of the uninsured. When comparing only the low-premium group with the uninsured, the difference in delayed care was more statistically pronounced (p = 0.05). Similarly, compared to the uninsured, children under a low-premium or commercial plan were less likely to forego care when ill (14.3% and 11.5% vs. 24.5%, p = 0.009). Because the three groups of children were generally healthy and similar in baseline health, the differences in health-seeking behaviors were likely attributable to the security afforded by insurance. However, as indicated by self-reported 12-month outpatient visitation data, care utilization patterns did not differ among the three groups (p = 0.796, Table 4). This observation suggests that the existing insurance schemes did not translate perceived improvements in access and affordability into improved care utilization, because most schemes did not alleviate the financial burden associated with routine care. On a positive note, overall satisfaction with healthcare was significantly higher among parents of an insured child than their uninsured counterparts (p = 0.01), with no marked difference between the low-premium and commercial insurance groups.

### Barriers to Enrollment

To further understand barriers to enrollment to and sustainability for cooperative programs, we probed parental concerns regarding children's insurance specifically and the existing insurance system in general. Table 5 shows that parents of an insured child were three times as likely to be positive about the insurance system as those of an uninsured child (48% vs. 16%), and that they were much less likely to be dissatisfied (13% vs. 31%). The difference suggested that direct experience with health insurance reinforced a better understanding and more positive opinion of the insurance system. However, leading concerns about insurance were rather similar among the three groups. High cost, followed by limited benefits, was the leading concern among over half of parents whose children either were uninsured or participated in a low-premium scheme. Although over half of the uninsured group were willing to enroll if the cost was low enough (58%) or if benefits improved (50%), only 3.7% in this group viewed health insurance as a necessity, underlining some fundamental barriers to insurance enrollment. Lack of knowledge about insurance appeared to be another barrier. Among those whose child was covered under a low-premium scheme, 4.3% indicated a lack of insurance knowledge; but this rate was three times as high among parents with an uninsured child. Distrust of business practice, lack of government oversight, and poor service quality were among other parental concerns about the insurance system.

**Table 5:** Surrogate Barriers to Insurance

**Percentage (Number) of Respondents Identifying the Barrier**	**Commercial**	**Low-premium**	**Uninsured**	**p-value^2^**
**Leading Barriers to Insurance**^1^	n = 61	n = 92	n = 108	
High costs	54.1%(33)	53.3%(49)	59.3%(64)	0.66
Limited benefits	22.9%(14)	21.7%(20)	16.7%(18)	0.53
Lack of knowledge on insurance	8.2%(5)	4.3%(4)	12.0%(13)	0.15
Distrust of business practice	8.2%(5)	7.6%(7)	8.3%(9)	0.98
Poor service quality	9.8%(6)	6.5% (6)	7.4%(8)	0.75
Lack of government regulation	3.3%(2)	5.4% (5)	8.3%(9)	0.40
Complex procedure	3.3%(2)	9.8% (9)	4.6%(5)	0.18

**Satisfaction with Insurance**	n = 100	n = 135	n = 141	<0.001
Satisfied	48.0%(48)	48.1%(65)	16.3%(23)	
Neutral	41.0%(41)	37.8%(51)	53.2%(75)	
Dissatisfied	11.0%(11)	14.1%(19)	30.5%(43)	

1. Respondents may choose multiple barrier items

2. Difference between the insured and uninsured groups tested via Chi-squared statistic

## Discussion

This survey shows that health insurance coverage for schoolchildren in Pinggu had risen in two waves, from 14% in 1999 to 44% in 2003, and to 54% in 2005. Commercial policies were the main vehicle prior to 1999; SSHI drove the first wave during 2000–2003; CHCF and RCMS later became major players, nearly doubling insurance rate among farmers' children. Although greater than a 1997 estimate of 20%[8] and a 2003 estimate of 43%[4], the current coverage rate of 54% remained low. Because of the high costs, commercial schemes remained unaffordable for most low and moderate income households, especially farming households. As a result, farmers' children largely depended on low-premium and cooperative schemes for insurance coverage that does not provide benefits for routine healthcare and are also low in reimbursement for covered medical events. While cooperative schemes such as RCMS and CHCF are becoming the principle insurance vehicle for schoolchildren, overlapping among these low-premium schemes in coverage, benefits, cost-reimbursement structure forces enrollees to choose one scheme over another. This was likely one reason why only 10% of insured farmers' children were under the RCMS, compared with 51% in the SSHI and 14% in the CHCF.

Perceived affordability played a delicate role in purchasing schoolchildren's health insurance. It is puzzling that those who appeared least or most able to afford insurance were more likely to enroll than their counterparts who were somewhat able to afford insurance. One explanation is that the somewhat-affordable may feel that the limited benefit options were unworthy of the premium even if it is low, whereas the unaffordable may value the basic protection against catastrophic events. This explanation echoes the argument of Chernew *et al*.[17] that universal coverage may not be achievable by reducing premiums alone. A recent study of villagers in Guizhou province China reports that 29% of the participants did not enroll in RCMS even when given a subsidy for the premium[18]. Low premiums may make insurance schemes more affordable, but narrow benefits may make them less practical, thereby dampening consumers' willingness-to-pay. For enrollees in cooperative schemes, substantial out-of-pocket co-payments have been found to be necessary in order to sustain the programs[19], thus adversely affecting healthcare access and diminishing the value of insurance policies[19,20]. Findings from this study reflected this phenomenon.

Our analysis suggests that by affording the enrollees a sense of security, the existing insurance schemes had improved perceived care access and affordability, and had also reduced delayed or forgone care. These improvements among those with a low premium policy over those uninsured were especially attributable to having insurance because insurance enrollment was neither driven by poor health nor promoted by a low premium. Insured children did not utilize more outpatient care than uninsured children, however, confirming that the existing insurance schemes did not alleviate the financial burden for routine care, and were ineffective in improving overall affordability. This argument is further supported by our findings that large portions of the insured under a low-premium scheme remained less positive about their access to and affordability of healthcare (57% and 42%, respectively).

The World Bank reported that in 2003 total contribution to the new RCMS from all sources covered only 20% of total household healthcare spending among enrolled Chinese farming households[5]. If enrollment to the cooperative schemes remains low, the programs may face adverse selection among enrollees and a shrinking pool of funds, which could threaten program sustainability and expansion[5,21]. Thus improving tangible benefits is essential for sustaining and expanding enrollment. A recent analysis argued that better benefits and reimbursement with more government funding are necessary for the RCMS to sustain in less developed rural areas[19]. A second study found that a considerable number of urban residents (24%) were actually willing to buy a commercial policy to compensate for outpatient care expense[22]. Still another study showed that both willingness-to-pay and actual amount contributed for enrolling to the BHIS increased with added benefits[23]. Offering more benefit options with flexible premiums within the existing cooperative programs would allow consumers to choose policies to fit their needs and increase willingness-to-pay, thereby boosting program enrollment.

We observed that compared to their uninsured counterparts, parents themselves insured were an order of magnitude more likely to enroll their children, and once enrolled were two times more satisfied with the insurance system. In a study of U.S. parents, Guendelman and Pearl[24] also observed that positive parental experiences with and improved knowledge about health insurance system promoted children's access to insurance. It is likely that consumers' experience with and perception about health insurance reinforce one another, and adequate knowledge about insurance promotes positive experience and mediates perception. Thus, community outreach could be an effective means for educating parents about children's health insurance, therefore promoting children's insurance enrollment.

There is currently no national health insurance program designated for schoolchildren, making them vulnerable in securing access to healthcare. In response to this systemic gap, regional programs have been emerging in parts of China, forming essentially a second tier of schemes to cover schoolchildren. However, vast disparities in regional economic development and variations in healthcare needs underscore the gap between the existing monolithic system and variable healthcare needs. To address this challenge requires innovative strategies on the part of the government and industry. One feasible approach is to expand the second-tier regional programs such as the CHCF, in conjunction with commercial programs to supplement the national schemes.

## Conclusion

The overwhelming choice of cooperative and low-premium insurance schemes among farmer's children reflected both their need for protection against major medical events and their willingness-to-pay or their affordability. Although these cooperative schemes did not fully meet schoolchildren's healthcare needs, especially with respect to routine care, they nonetheless positively impacted on perceived access to and affordability of healthcare, reduced undesirable health-seeking behaviors, and improved overall satisfaction with healthcare.

To increase the tangible value of existing health insurance programs, it is both necessary and feasible to offer more insurance options through expanding the national programs such as the RCMS or by developing second-tier, regional programs such as the CHCF to help cover routine healthcare needs.

Government should play a central role in funding and guiding national and regional health insurance programs, while simultaneously strengthen regulation of the health insurance market. Improved government oversight is not only high in consumer demand, but also will enhance consumer confidence in the healthcare system.

Improving parental knowledge about health insurance can help increase schoolchildren's insurance enrollment. The success of SSHI, by means of partnerships with school administrations, demonstrates that community outreach can be a highly effective marketing tool in educating the parents about children's health insurance.

Despite the small scale and specific scope of this study, our findings are relevant on a much larger scale, as Pinggu represents a large segment of Chinese rural/suburban townships. Findings from this study fill an important information gap for schoolchildren, are useful in guiding future evaluation of health insurance coverage, but need to be replicated on a larger scale. Evaluation of China's evolving healthcare needs and healthcare outcomes should be conducted on an ongoing basis. Integrating the evaluation of schoolchildren's insurance into this process by utilizing national resources such as the National Health Services Survey appears both attractive and feasible.

## Competing interests

The authors declare that they have no competing interests.

## Authors' contributions

JMZ participated in the conception, design, and conduct of this study. She led the efforts in the development of instruments, data collection, analysis, and draft of the paper. YZ participated in the conception and design of the study, development of the instruments, conducting data analysis, and the writing of the article. RL participated in the design, implementation, and conducting of the study. All read and approved the content of this paper.
